# Retained capsule in covert strictures at the stoma closure site of an ileal pouch, retrieved following endoscopic strictureplasty via retrograde enteroscopy: a case report

**DOI:** 10.1016/j.igie.2025.11.001

**Published:** 2025-11-17

**Authors:** Shanshan Wang, Dana J. Lukin, Bo Shen

**Affiliations:** 1 The Global Center for Integrated Colorectal Surgery and IBD Interventional Endoscopy, Columbia University Irving Medical Center/New York Presbyterian Hospital, New York, New York, USA; 2 Jill Roberts Center for Inflammatory Bowel Disease, Gastroenterology and Hepatology, Department of Medicine, New York Presbyterian/Weill Cornell Medical Center, New York, New York, USA

## Abstract

Video capsule endoscopy (VCE) is an important tool to evaluate small-bowel involvement in inflammatory bowel disease. However, the risk of capsule retention increases in the presence of strictures or obstruction. In patients with total proctocolectomy and ileal pouch-anal anastomosis (IPAA), the use of VCE is challenging because of the surgically altered anatomy and the common presence of strictures. A 46-year-old man with a history of ulcerative colitis and total proctocolectomy with IPAA underwent VCE to assess persistent, unexplained anemia, which impaired his training for a marathon. The capsule was retained at a high stoma closure site with 2 severe strictures undetected on magnetic resonance imaging and previous routine pouchoscopy. Endoscopic balloon dilation and endoscopic strictureplasty were performed with eventual retrieval of the capsule via retrograde enteroscopy. A thorough endoscopic evaluation of the ileal pouch, including the stoma closure site, is crucial prior to VCE.

## Case description

A 46-year-old man with a history of ulcerative colitis who underwent proctocolectomy and ileal pouch-anal anastomosis (IPAA) presented with iron deficiency anemia. His pouch-related history included an episode of small-bowel obstruction in 2022, with a computed tomography (CT) scan showing bowel dilation proximal to the stoma closure site. However, a follow-up abdominal-pelvic magnetic resonance imaging (MRI) with enterography, performed 4 months later, showed decreased small-bowel dilation, with no evidence of obstruction. He also had an anopouch stricture treated endoscopically in 2023. The patient reported using oxaprozin, a nonsteroidal anti-inflammatory drug (NSAID), for 30 days in the month preceding symptoms onset, for epididymitis. The patient had no other relevant medical, family, or social history. Clinical symptoms included asthenia, heartburn, and dyspepsia, without overt gastrointestinal bleeding. Physical examination results were unremarkable. In 2024, laboratory results showed hemoglobin 9.1 g/dL (13.2-17.1 g/dL), mean corpuscular volume 81.4 fL (80.0-100.0 fL), ferritin 6 ng/mL (38-380 ng/mL), iron saturation 4% (20%-48%), and normal vitamin B12 and fecal calprotectin. Esophagogastroduodenoscopy revealed peptic duodenitis and chemical gastropathy, likely NSAID-induced; *Helicobacter pylori* immunostaining was negative. Pouchoscopy showed an anal stricture, treated with digital dilation, and 2 subcentimeter ulcers in the prepouch ileum. The patient started on a proton pump inhibitor (PPI) and received 2 rounds of iron infusions. However, hemoglobin remained low (9.6 g/dL) after 3 months. PPI was tapered as symptoms improved.

**Figure 1:**
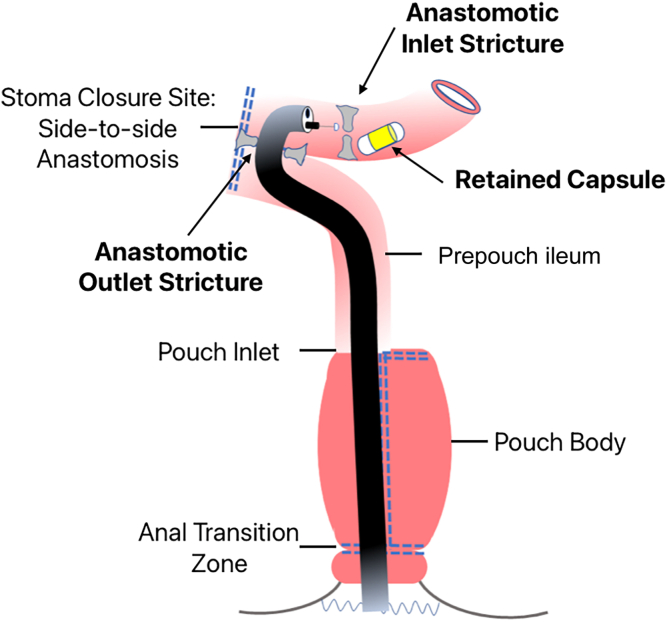
A diagram with anatomical landmarks and surgical staple lines (*blue dashed lines*). The retained capsule was visualized after endoscopic balloon dilation and endoscopic strictureplasty in the inlet stricture of the side-to-side anastomosis, using a slim colonoscope.

**Figure 2:**
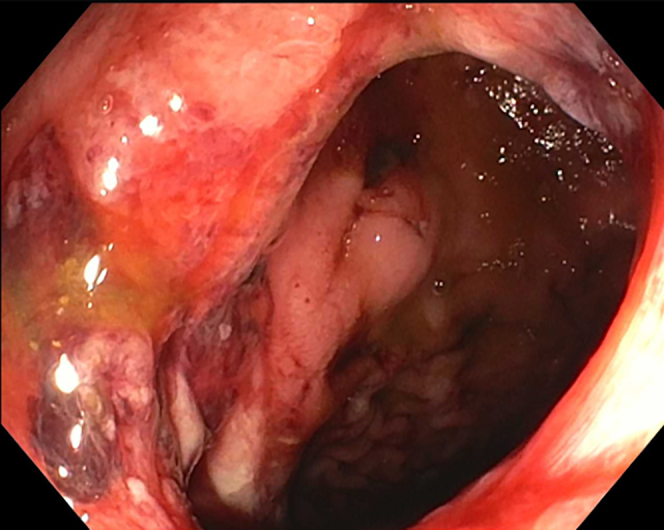
A severe, not traversable stricture at the outlet of the side-to-side anastomosis, treated with endoscopic balloon dilation, after which the colonoscope was passed without resistance.

**Figure 3:**
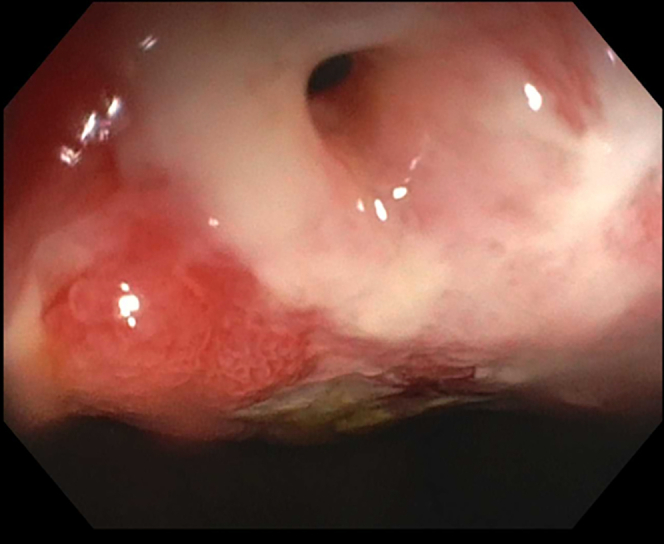
A pinhole stricture covered with white fibrin, not traversable with a colonoscope, at the inlet of the side-to-side anastomosis of the diverted loop ileostomy.

**Figure 4:**
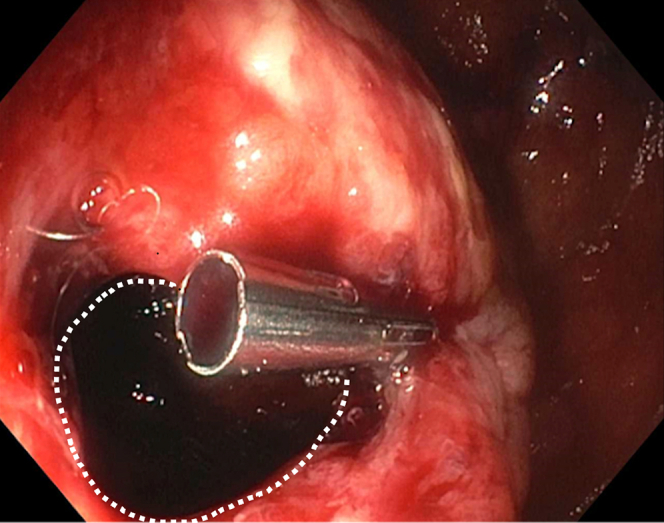
Anastomotic inlet stricture treated with endoscopic balloon dilation and endoscopic strictureplasty using an insulated-tip knife and an endoscopic clip placed to maintain lumen patency (*outlined*).

**Figure 5:**
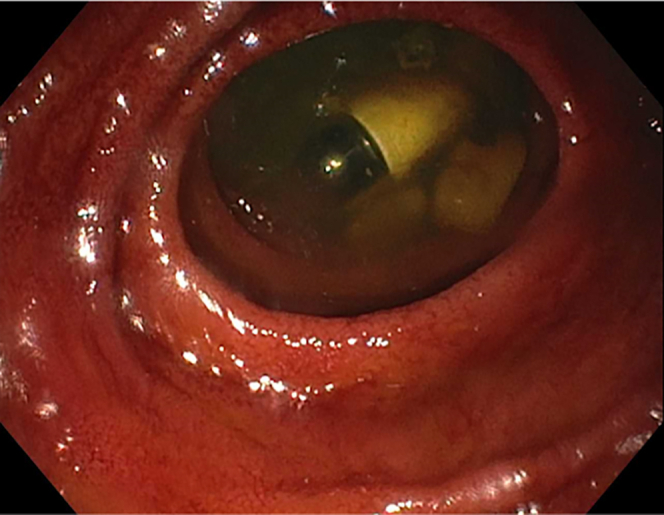
Retained capsule in the prepouch ileum, beyond the anastomotic inlet stricture, identified after endoscopic stricture therapy.

**Figure 6:**
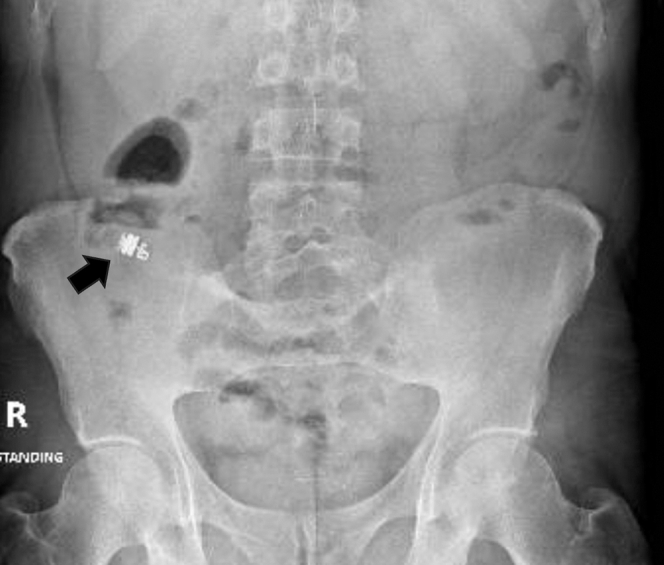
Abdominal radiograph performed after endoscopic stricture therapy showed the capsule retained in the small bowel located in the low-right quadrant (*black arrow*).

**Figure 7:**
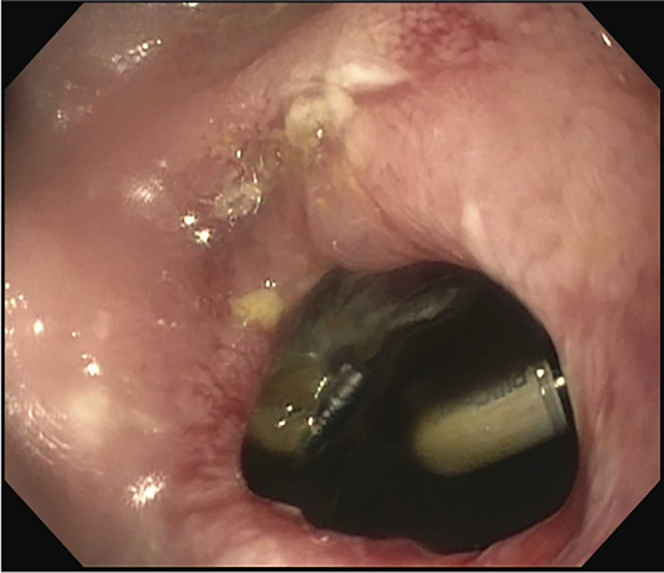
On the retrograde enteroscope, the capsule had migrated into the cavity of the side-to-side anastomosis, between the treated inlet and outlet strictures, which resembled a pseudodiverticulum.

**Figure 8:**
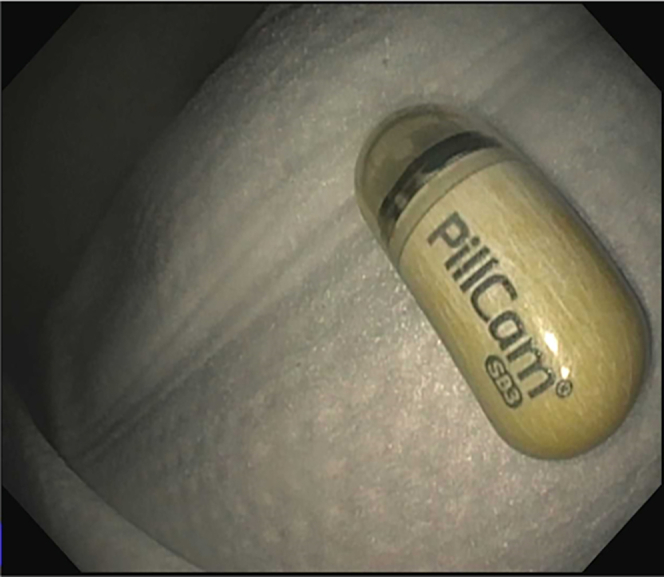
Removed capsule.

To further evaluate the etiology of anemia, video capsule endoscopy (VCE) was administered with no patency capsule, given the absence of a small-bowel stricture on MRI. Endoscopic retrieval was planned as a backup for the known anal stricture. However, the capsule failed to pass after 2 weeks. A pouchoscopy was advanced 70 cm from the anus, without identifying the retained capsule. In addition, a solitary 10-mm ulcer with oozing blood was found in the pouch body. Repeat pouchoscopy 1 week later with a slim colonoscope (PCF-H190TL; Olympus America, Center Valley, Pa, USA) revealed 2 strictures at the prior side-to-side anastomosis of the diverting loop ileostomy, located at 50 cm proximal to the pouch inlet (Fig. 1). The first stricture, at the anastomotic outlet, measured 10 mm × 7 mm (length × diameter), was not traversable, and was treated with endoscopic balloon dilation to 15 mm (Fig. 2). The second, at the inlet, was a 10-mm × 2-mm pinhole ulcerated stricture and was treated with endoscopic balloon dilation to 12 mm, followed by endoscopic strictureplasty using an insulated-tip knife (Olympus America) and an endoscopic clip to maintain luminal patency (Figs. 3 and 4).^1^ The colonoscope was then traversed without resistance. The neoterminal ileum above the stoma closure site revealed small ulcers and food debris. The retained capsule was visualized beyond the anastomotic inlet stricture but was not retrievable because of angulation of the scope and mobile VCE (Fig. 5). Three weeks later, with no spontaneous passage of the capsule confirmed by a plain abdominal radiograph (Fig. 6), an enteroscopy via the anus (SIF-H190; Olympus America) was performed. The capsule had migrated into the cavity of the stoma closure site, which resembled a pseudodiverticulum (Fig. 7), and was successfully removed (Fig. 8) using a Roth Net (model 360; Steris Corporation, Mentor, Ohio, USA). The previously treated strictures were patent, with otherwise normal pouchoscopy. No adverse events were noted. Currently, the patient's symptoms and anemia have resolved, and he expressed satisfaction with the recovery.

## Discussion

Inflammatory bowel disease after IPAA is frequently associated with inflammatory, structural, and metabolic issues.^2^ Anemia represents a metabolic sequela in pouch patients, the pathogenesis of which appears multifactorial yet poorly defined, with VCE being helpful in the identification of the source.^3^ VCE is a noninvasive diagnostic procedure of the small bowel, with capsule retention reported in 1% to 13% of cases, which further increased in small-bowel stenosis compared with obscure gastrointestinal bleeding.^4^ Although most retained capsules are asymptomatic and managed conservatively, prolonged retention may lead to obstruction or perforation, requiring endoscopic or surgical intervention.^5^

Pouch strictures are frequent issues after IPAA, occurring in up to 38%.^6^ Common sites include the pouch inlet, pouch-anal anastomosis, and stoma closure site. Multiple factors were identified, encompassing surgery-related factors, the use of NSAIDs, and Crohn’s disease (CD) of the pouch.^7^ In patients with an ileostomy, isolated strictures without inflammation in other segments of prepouch ileum are likely related to surgical factors—such as ischemia or anastomotic tension—rather than CD of the pouch.^7^ The use of NSAIDs has also been linked to pouchitis.^8^ In our patient, the stricture location and lack of inflammation elsewhere after cessation of NSAIDs suggest a surgery-related stricture, although NSAIDs may have exacerbated the condition.

Accurate characterization of pouch structural disorders often requires both endoscopic and radiologic evaluation, including fluoroscopic defecography, CT, and MRI.^7^ However, even MRI may fail to detect a stricture, particularly during asymptomatic periods, as demonstrated in our patient. Although sometimes technically challenging, a thorough endoscopic evaluation of pouch anatomical landmarks—including stoma closure site, prepouch ileum, inlet, pouch body, cuff, and anal transition zone—is crucial, especially before planning VCE. Unlike most patients, whose stoma closure site is located at 20 to 25 cm above the pouch inlet,^6^ our patient presented an unusually high stoma closure site, located 50 cm above, which required a slim colonoscope for access and an enteroscope for the capsule retrieval. Patency capsules, dissolvable capsules mimicking the actual device, have been used to select patients with stenosis to perform VCE;^9^ however, their role in pouch patients remains unclear.

This case illustrates NSAID-induced gastropathy, enteritis, and pouchitis, complicated by persistent iron deficiency anemia and radiologically occult strictures. The strictures at the stoma closure site, susceptible to NSAID-related injury, may have contributed to delayed hemoglobin recovery, ultimately leading to VCE use and capsule retention. Although limited by being a single case, it underscores the need for caution when considering VCE in pouch patients.

In summary, comprehensive endoscopic evaluation of the pouch, including the stoma closure site, is essential before VCE. The pouch scope should be advanced beyond the level of the stoma closure site to fully assess for structural abnormalities.

## Ethical approval

Data were extracted from our prospectively maintained and Institutional Review Board–approved database (AAAN 3966) at Columbia University Irving Medical Center/NewYork–Presbyterian.

## Patient consent

Electronic medical records were collected for patients who had not withdrawn research authorization to complete clinical and diagnostic data collection.

## Disclosure

The following authors disclosed financial relationships: B. Shen: Consultant for Janssen; research/education grants from AbbVie, Takeda, and GIE Medical. D. J. Lukin: Consultant for AbbVie, Altrubio, Boehringer Ingelheim, BMS, Johnson & Johnson, Palatin, Pfizer, Prime, PSI, Takeda, and Vedanta; speaking fees from AbbVie and Johnson & Johnson; research grants from Boehringer Ingelheim and Johnson & Johnson. S. Wang disclosed no financial relationships.
